# The Royal Sea-Bathing Hospital, Margate

**Published:** 1902-07-05

**Authors:** 


### THE ROYAL SEA-BATHING HOSPITAL, MARGATE.

### THE YEAR'S WORK.

The total number of patients admitted during 1901 was 545; the same number was discharged, and of these, 154 were men, 159 women, 126 boys, and 106 girls. Many applications were received for the admission of children under six, but only a few could be taken in, as no suitable accommodation exists. "This," says the report, "is greatly to be regretted, since the possibilities of cure are far greater in early life. Many of the cases returned as unbenefited would doubtless have been cured had the patients been admitted when the disease was in its initial stage." One hundred and six patients remained in the hospital at the close of the year, making a total of 651. Ten patients were "ailing *nothing*" on admission; they were practically convalescent from recent tuberculous disease when they arrived. Many of the cases returned as unbenefited were received in the last stage of the disease, and the honorary visiting surgeons, in their report, remark: "It is to be hoped that medical men may appreciate the waste of public money involved in treating such cases in a hospital specially adapted for the early treatment of early cases of disease, when so many institutions are available for the maintenance of incurables."

The structural alterations recommended in 1900 by the Medical Board have not yet been carried out for want of funds. The report for 1900 says that the Board recommended "two or three rotating tents for the use of patients, to be fixed on the roof;" also, that the construction of two wards on the ground at present unoccupied was advisable for the reception of patients in the early stages of phthisis, their opinion being that there were no wards which it would be desirable to appropriate to the purpose.

A trained theatre nurse has been appointed, and a sterilising apparatus has been procured and placed in her charge.

There is no allusion in the report for 1901 to the Finsen Light Treatment for Lupus, which was started in the previous summer; the report for 1900 stated that the results in some cases were good, but that improvement was not by any means universal. It was hoped that the second season might produce a larger proportion of satisfactory cases.

The large deficits of the past two years, amounting to £4,413 18s., and the consequent sale of stock, are causing the directors great anxiety; they state that the closing of some of the beds will be a necessity unless funds are forthcoming. An appeal is being made to King Edward's Fund for help, on the grounds that two-thirds of the patients come from London. In 1901 the Hospital Sunday Fund gave £291 13s. 4d., and the Saturday Fund £21. Patients' payments amounted to £1,550; subscriptions were £1,379; donations £1,244; and the total ordinary income was £5,903 9s. 11d. Legacies amounted to £814 9s. 3d. The total ordinary expenditure was £7,840; extraordinary (repairs, building improvements, etc.), £304 16s. 10d.

